# One-Pot Synthesis of Multi-Branch Gold Nanoparticles and Investigation of Their SERS Performance

**DOI:** 10.3390/bios8040113

**Published:** 2018-11-20

**Authors:** Weifeng Lv, Chenjie Gu, Shuwen Zeng, Jiaguang Han, Tao Jiang, Jun Zhou

**Affiliations:** 1 Institute of Photonics, Faculty of Science, Ningbo University, Ningbo 315211, China; lvweifeng.nbu@gmail.com (W.L.); guchenjie@nbu.edu.cn (C.G.); jiangtao@nbu.edu.cn (T.J.); 2 XLIM Research Institute, UMR 7252 CNRS/University of Limoges, 123, Avenue Albert Thomas, 87060 Limoges CEDEX, France; 3 Center for Terahertz Wave, Key Laboratory of Opto-Electronic Information Science and Technology, Ministry of Education, College of Precision Instrument and Optoelectronics Engineering, Tianjin University, Tianjin 300072, China; jiaghan@tju.edu.cn

**Keywords:** gold nanoparticle with branches, one-pot synthesis, LSPR frequency tuning, surface enhanced Raman scattering

## Abstract

Gold nanoparticles with multiple branches have attracted intensive studies for their application in sensing of low trace molecules. A large number of the merits found on the gold nanoparticles for the above applications are attributed to the strong localized surface plasmon resonance excited by the incident radiation. However, a facile and flexible way of synthesizing the multi-branch gold nanoparticles with tunable localized surface plasmon resonance frequency is still a challenge for the plasmonic research field. Herein, we report an efficient one-pot synthesis of multi-branch gold nanoparticles method that resembles a seed-medicated approach while using no further chemicals except chloroauric acid, ascorbic acid and 4-(2-Hydroxyethyl)-1-piperazinyl]-ethanesulfonic acid. By controlling the amounts of ascorbic acid volumes in the reaction mixture, the morphology and the localized surface plasmon resonance frequency of the synthesized multi-branch gold nanoparticles can be manipulated conveniently. Moreover, using the 4-Mercaptobenzoic acid as the Raman reporter, the multi-branch gold nanoparticles show superior surface-enhanced Raman spectroscopy characteristics that can be potentially used in chemical and biological sensing.

## 1. Introduction

Gold nanoparticles have attracted intensive studies for their application in biological sensing, immunoassay and food safety [1,2,3,4,5]. Plenty of the merits found on the gold nanoparticles for the above applications are ascribed to the strong localized surface plasmon resonance (LSPR) excited on their surface by the incident radiation. Moreover, the fascinating arts of the gold nanoparticles are the tunable LSPR frequency for different application scenarios through the size, shape and even composition engineering [6,7,8].

Surface-enhanced Raman scattering (SERS) phenomenon observed on nanoparticles results in enhanced Raman scattering signals of the molecules that are chemically bonded or physically adsorbed on them [9]. The principle of the SERS is usually attributed to two well-known mechanisms, i.e., electromagnetic mechanism (EM) and chemical mechanism (CM). EM originates from the enhanced localized electromagnetic field near the nanostructure surface produced by the LSPR, while the CM results from the charge transferring between the molecules and nanoparticles/substrate [10]. Both of these two mechanisms induce the boosted polarizability of the target molecules and, consequently, promote the Raman scattering signal [11,12]. In view of these fundamentals, many works have been carried out to improve the near-surface electromagnetic field intensity or charge transferring efficiency [13,14,15,16]. In recent studies, much attention has been placed on tuning nanoparticle shape-dependent LSPR properties to achieve intense localized electromagnetic field [17,18].

Different types of gold nanoparticles, such as gold nanorod, nanoflower, nanopolyhedral and even nanourchin, etc. have been extensively investigated for their easy-tuning LSPR frequency and superior SERS performance [19,20,21,22,23]. Meanwhile, studies on the gold nanoparticles with multi-branches (AuNBr) also reveal their excellent SERS activities [24,25,26,27,28]. Using the 4-(2-Hydroxyethyl)-1-piperazinyl]-ethanesulfonic acid (HEPES) as the reducing and capping agent, the shape and length of the branches of the AuNBr are able to be conveniently adjusted by controlling the HAuCl_4_/HEPES ratios, the temperature or surfactant [17,29,30,31,32,33]. However, many studies have shown that the mechanism of synthesizing the AuNBr is a kind of two-step procedure [21,25,27,30]. Generally, in the first step, a small amount of HAuCl_4_ is added into HEPES solution to facilitate the formation of Au seeds or in certain experiments, pre-prepared Au nanoparticles is used straightforwardly as seeds, thereafter, more HAuCl_4_ is added into the reaction mixture to further promote the anisotropic growth of the branches on the Au seeds. However, due to the low reduction capability of the HEPES, the typical size of the non-seed mediated nanoparticle by using the HEPES usually is less than 30 nm, and the whole synthetic procedure takes more than one hour [18,25,27,32].

Herein, we report a one-pot synthesis of AuNBr that resembles seed-medicated approaches while using no further toxic chemicals except HAuCl_4_, ascorbic acid (AA) and HEPES. In this synthetic procedure, the core sizes (from 26 nm to 50 nm) and branches (from 7 nm to 10 nm) of the nanoparticles are able to be easily tuned by the amounts of the added AA volumes, while the whole synthesis duration is less than 40 min, and from the optical characteristics of the synthesized AuNBr, it shows up to 84-nm LSPR change. Moreover, by using the 4-Mercaptobenzoic acid (4-MBA) as the Raman reporter and finite element method (FEM) simulation, the SERS activities of these AuNBr are experimentally and theoretically investigated; the results indicate that a maximum Raman enhancement factor of 1.3 × 10^8^ can be achieved, which is assured by the significantly enhanced near surface electromagnetic field on the optimized AuNBr morphology. Overall, this one-pot synthesis method provides a facile and efficient method to prepare the AuNBr, which can be potentially used for bio-sensing.

## 2. Materials and Methods

### 2.1. Chemicals

Tetrachloroauric (III) acid trihydrate (HAuCl_4_·3H_2_O, 99.5%) were purchased from Sigma-Aldich. Sodium hydroxide (NaOH) were purchased from Aladdin. 2-[4-(2-Hydroxyethyl)-1-piperazinyl]-ethanesulfonic acid (HEPES) were purchased from Macklin. Ascorbic acid (AA) was purchased from Bodi Chemical Reagent Co. (Tianjin, China). 4-MBA was obtained from J&K Chemical. Milli-Q water (18.2 MΩ·cm^−1^ resistivity) was used for all solution preparation. All glassware was cleaned using aqua regia (Caution! aqua regia is highly corrosive and should be handled with great care) and rinsed with deionized water several times before the experiments.

### 2.2. Synthesis of AuNBr

Aqueous stock solution of HEPES with a concentration of 100 mM was prepared with the Milli-Q water, and the pH value was adjusted to 7.4 at 4 °C by adding 1 M NaOH solution. Aqueous stock solution of AA with a concentration of 100 mM was also prepared with Milli-Q water. In a typical experiment, 1 mL of 100 mM HEPES (pH = 7.4) was mixed with 9 mL of deionized water in the beaker, and totally eight sets of solutions were prepared. Thereafter, different volumes of 100 mM AA solution (10 μL, 20 μL, 30 μL, 40 μL, 50 μL, 60 μL, 70 μL, 80 μL) were drop-wisely added into each beaker, respectively. In the final step, 0.3 mL of 20 mM HAuCl_4_ was added into the reaction mixture. With gentle shaking, these reaction mixtures were maintained for 40 min at 4 °C. In all of these reactions, the amount of added AA was insufficient to fully consume the HAuCl_4_, and the rest of HAuCl_4_ after reacting with AA was finally depleted by the excess HEPES in the mixture.

### 2.3. SERS Measurement

The SERS performances of the AuNBr were evaluated with 4-MBA Raman molecules. Firstly, the sample solutions were prepared by adding 20 μL of 10 mM 4-MBA solution to the above purified 3 mL of AuNBr solutions under stirring, and the resultant solutions were agitated for 5 h. Afterwards, the mixtures were centrifuged at 10,000 rpm for 20 min to remove unbound 4-MBA molecules, then the sediments at the bottom of centrifuge tubes were re-dispersed in 3 mL deionized water as 4-MBA AuNBr solutions, and transferred to the quartz cuvette for SERS measurement.

### 2.4. Characterization

SU-70 FESEM (Hitachi, Tokyo, Japan) instrument was used to collect the scanning electron microscopy (SEM) images under an accelerating voltage of 5 kV. Transmission electron microscope (TEM), high-resolution transmission electron microscope (HRTEM) images were obtained with TEM (JEM-2100F, JEOL, Tokyo, Japan) operated at accelerating voltage of 200 kV. UV–vis absorption spectra were monitored with a spectrometer (TU1901, P-General, Samutprakarn, Thailand). SERS measurements were carried out on a Raman microscope (BWS415, B&W Tek Inc., Newark, DE, USA) by using a 785 nm semiconductor laser as the excitation source. The Raman spectra were collected under the laser power of 10 mW, and integration time of 10 s.

### 2.5. Numerical Simulation

Finite element method (FEM) simulation was performed by using the COMSOL Multiphysics software (COMSOL AB, Stockholm, Sweden). The AuNBr model structures were created based on the TEM images. Generally, the model consisted of the superposition of the spherical core and random distributed branches, in which the branches were approximated with the spheroids. The physical profile of the core and branches were obtained by measuring the parameters from the corresponding AuNBr TEM image. In the theoretical modeling, perfect matched layer (PML) was used, and plane wave with the wavelength from 400 nm to 800 nm was exerted as the excitation source, the absorption spectra and the near surface electromagnetic field were recorded.

## 3. Results

### 3.1. The Effect of the Added AA Volumes

The up-to-date methods of preparing the AuNBr by using the HEPES usually comprise two approaches. One is the Au seeds mediated branches growth, in which the HEPES is used as the reducing and shape-directing agent; the other one first adding small amount of HEPES into the HAuCl_4_ solution to prepare the Au seeds, then more HEPES is added into the reaction mixture to further promote the anisotropcal growth of the AuNBr. Both of them are a two-step synthetic procedure. Nevertheless, in these experiments, the controlling of AuNBr morphologies as well as the time efficiency are big challenges. Herein, a one-pot synthesis of tunable AuNBr that resembles seed-medicated approaches is discussed, and chemicals of only HAuCl_4_, AA and HEPES are used. In order to properly control the morphology of the AuNBr, the amounts of AA that used during the reaction are investigated. By implementing the aforementioned synthesizing method, eight samples with different added amounts of AA volumes (10 μL to 80 μL) are examined.

**Figure 1:**
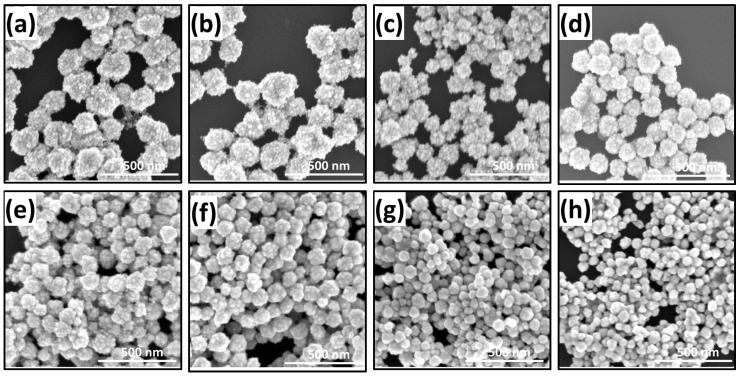
FESEM images of the AuNBr synthesized with different amounts of AA volumes: (**a**) 10 μL; (**b**) 20 μL; (**c**) 30 μL; (**d**) 40 μL; (**e**) 50 μL; (**f**) 60 μL; (**g**) 70 μL and (**h**) 80 μL.

**Figure 2:**
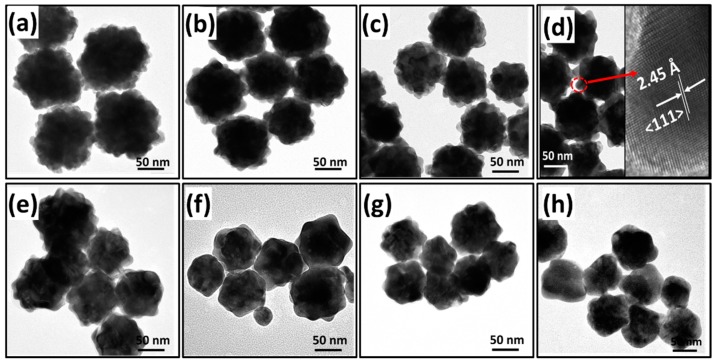
TEM images of the AuNBr synthesized with different amounts of AA volumes: (**a**) 10 μL; (**b**) 20 μL; (**c**) 30 μL; (**d**) 40 μL; (**e**) 50 μL; (**f**) 60 μL; (**g**) 70 μL and (**h**) 80 μL.

The typical FESEM (Field Emission Scanning Electron Microscopy) and TEM images of the AuNBr are shown in the Figure 1 and Figure 2, respectively. It could be observed on the Figure 1 that the as-synthesized nanoparticles are quasi-spherical, and branches protrude outwards on the nanoparticle surface. Moreover, when the added AA volumes increase, it shows that both the size of the AuNBr core and the number of the branches on the surface decreased. These evidences indicate that the amount of the added AA volumes has a significant effect on the particle morphology. The TEM images (Figure 2) of the AuNBr obtained with different AA volumes reveal more structural information, and details are listed in Table 1. It can be found on the images that the nanoparticles show typical structures that branch and are randomly distributed on solid core AuNBr. The branches are obtuse and exhibit varying contrast which is different from the uniformly dark solid core. Moreover, it can be found that when the added AA volumes are less than 60 μL, branches where clear profiles can be observed, whereas when the added AA volumes increase to 60 μL or even higher volumes, e.g., 70 μL and 80 μL, the branches on the surface gradually fade, and in the case of 80 μL, the nanoparticle is more or less like the regular Au nanoparticle. More specifically, the mean core size of the AuNBr shrinks from 50 nm to 25 nm when the added AA volumes increase from 10 μL to 80 μL, while for the branches, the area of the foot region expands and the total length reduces. These observations clearly illustrate that the AuNBr evolves from relatively large core with dense and tiny branches to small core with sparse and hypertrophic ones. Additionally, the high-resolution image for the case of 40 μL AA is also shown in the inset of Figure 2d. It is clearly illustrated that the branches on the AuNBr is single crystalline with the lattice constant of 2.45 Å, which is consist with the other experiments [27].

**Figure 3:**
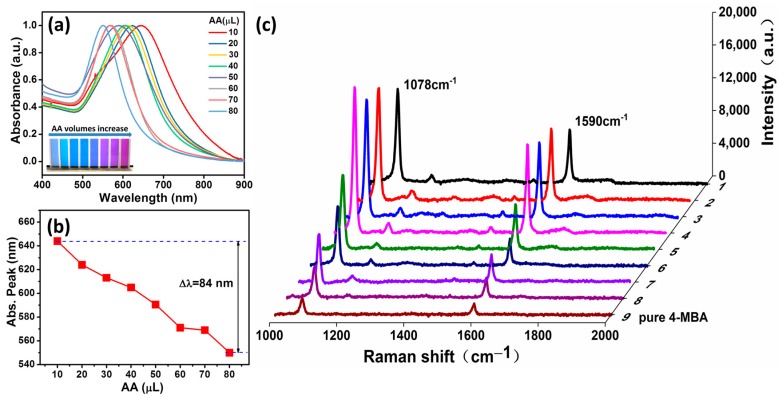
(**a**) UV–vis absorption spectra of AuNBr colloidal prepared with different amounts of AA volumes. The inset shows the true color of prepared AuNBr colloid; (**b**) tunability of the absorption peaks with the change of the AA volumes; (**c**) the SERS spectra of 4-MBA adsorbed on the AuNBr. Labels 1–8 in the y axis correspond to the added AA volumes from 10 μL to 80 μL, respectively.

To further investigate the optical characteristics of the AuNBr, the photographic images and UV–vis spectroscopy of the synthesized AuNBr are measured and shown in Figure 3a. As it can be obviously found that distinguished color change of the solution from slate blue to violet red as the amount of the added AA volumes increase. In addition, for the UV–vis spectroscopy, the absorption peak for the case of added AA volumes equal to 80 μL is centered at 550 nm, which is quite close to the absorption peak of regular Au nanoparticle. With the simultaneous increase of the core size and the branches length, the absorption peak shifts from 550 nm to 644 nm, which gives a total wavelength shift of 84 nm (Figure 3b).

### 3.2. SERS Performance of the AuNBr and Enhancement Factor

In order to assess the sensing capability of the AuNBr, the SERS signals are measured by using the 4-MBA (10 mM) as Raman reporter, which is shown in Figure 3c. The peaks at 1078 cm^−1^ and 1587 cm^−1^ that are assigned to ν(C-C) benzene ring-breathing modes are clearly shown. The SERS performance of the AuNBr is evaluated by calculating the enhancement factor (***EF***) of the nanoparticles, in which the following equation is used [7,34] (see the detailed numerical calculation in supplementary materials):

$$EF=(I_{SERS}\times N_{NR})/\left(I_{NR}\times N_{SERS}\right)$$

where $I_{SERS}$ and $I_{NR}$ are the integrated peak intensity at 1078 cm^−1^ from SERS and from the pure 4-MBA solution, respectively. $N_{NR}$ is the number of bulk molecules probed in bulk sample, and $N_{SERS}$ is the number of molecules that adsorbed on the nanoparticle. The calculated ***EF****s* of the nanoparticles are shown in Table 2, respectively. Referring to the ***EF****s* of the nanoparticle reported in other work [19,27,35,36,37], the AuNBr here shows comparable SERS performance, and the maximum ***EF*** is found on the AuNBr synthesized with 40 μL AA, which can reach 10^8^ at the excitation wavelength of 785 nm.

## 4. Discussion

**Figure 4:**
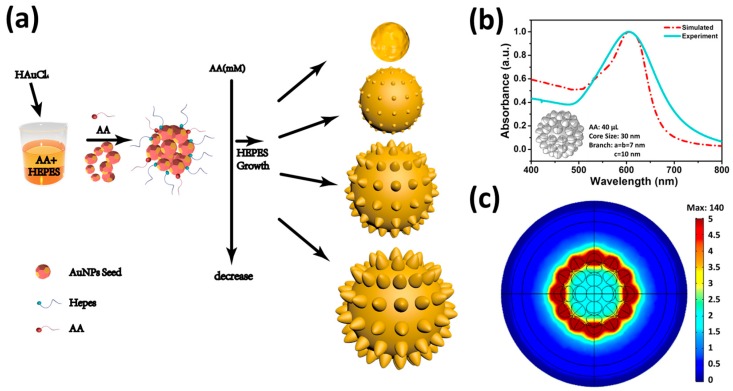
(**a**) Schematic plot of the growth mechanism; (**b**) structural model and the simulated absorption curve compared with the experimental one (AA = 40 μL); (**c**) near-surface electric field intensity of the simulated model.

Based on the above experimental results, it can be seen that the amounts of the added AA volume play a pivotal role in shaping the particle morphology, and this critical function could be attributed to the relatively strong reducing capability of the AA compared to that of HEPES. In Figure 4, a schematic plot of the growth mechanism is shown. As we can see that when HAuCl_4_ is dropped into the AA and HEPES reaction mixture, AA tends to react with the HAuCl_4_ firstly and forms the Au seeds instantaneously. However, in this event, due to the added amounts of AA volumes in each case being different (resulting in different AA concentrations in the reaction mixtures), and according to [38,39,40], we can have the inference that the nucleation rate of the Au^0^ is much faster when AA concentration is high, as a result, Au seeds of smaller size are synthesized. On the contrary, the reducing reaction is much slower when AA concentration is low, and Au^0^ tends to accumulate on the same nucleation, producing larger Au seeds. Thereafter, when the reaction between the HAuCl_4_ and AA is finished, the residual HAuCl_4_ continuously reacts with the excess HEPES. Since the by-product between the HEPES and HAuCl_4_ reaction favors to anisotropcally adsorb on the high-energy faces of the Au seeds (surface energy: (100) > (110) > (111)), and inhibits the growth of the Au on the high-energy faces, therefore, under the relatively low concentration of HAuCl_4_ reaction mixture, branches tend to grow on the (111) crystalline face of the Au seed [29]. Besides, it is worth noting that the foot region of the branch expands (a, b of the ellipsoid) and the length is reduced (c of the ellipsoid) when the AA volumes gradually increase (see Table 1). Since branches usually start to appear on the (111) surfaces, the initial surface energy for small cores is higher than that of large cores. Therefore, in order to lower the total surface energy, the foot region of the branch on the small core tends to expand laterally more intensely during the branch-growing phase, contributing to the core size. Meanwhile, the increase of branch length is retarded due to lack of Au^0^. In addition, the relatively small surface area of the core further limits the number of (111) faces that expose to the reaction mixture, and consequently limits the total number of branches on the surface. Thus, the above growth strategy ultimately results in spars, short and hypertrophic branches [25,41]. On the other hand, for large cores, the initial surface energy is relatively lower than that of small cores. The laterally expanding foot religion during the branch-growing phase is, therefore, much weaker, and the length of the branch increased more efficiently. Additionally, a higher number of (111) faces are exposed to the reaction mixture for the large cores, and more branches on the surface grow simultaneously. As a result, this growth strategy produces tiny, dense and long branches.

Moreover, nanoparticles with branches are known to exhibit hybridized surface plasmon characteristics that result from solid core and individual branches, respectively [25]. However, in our experiment, a single absorption peak with relative broad full width at half maximum (FWHM) can be observed except the one synthesized with 10 μL AA. This could be attributed to the branches on these AuNBr being relatively short, resulting in minor energy splitting between the bonding and anti-bonding state. For the AuNBr synthesized with 10 μL AA, the hump observed at short wavelength region could be ascribed to the relatively long and dense branches, which causes increased energy splitting during the hybridization. In addition, the absorption peaks of the nearly spherical Au nanoparticle synthesized with 20 μL AA is at 550 nm, and the increase of the core size of the AuNBr will red shift the peak positions, but only hybridizing with the absorption peak of the branches which locates at near infrared region that can achieve such significant LSPR frequency change [25].

Furthermore, the calculation of the ***EF****s* shows that all of the AuNBr synthesized with different amounts of AA volumes have relatively good SERS performance, nevertheless, the ***EF*** of AuNBr synthesized with 40 μL AA is still about two orders higher than that of the AuNBr synthesized with 80 μL AA, and ten times higher than that of the AuNBr synthesized with 10 μL AA. Although the absorption peak of the AuNBr synthesized with the reducing of added AA volumes is gradually shifted towards the wavelength of Raman exciting laser (785 nm), and consequently promoting the resonance Raman scattering, this does not explain the experimental observation that the maximum ***EF*** appears on the AuNBr synthesized with 40 μL AA. To shed light on the physical mechanism of this observation, the near-surface electric field intensity of the AuNBr is calculated by the FEM simulation, which is shown in the Figure 4c. It can be seen that the peak intensity of the electric field of the AuNBr synthesized with 40 μL AA reaches 140 eV/cm, which is the highest one among all of the AuNBr (see Figure S2 in supplementary). Considering the near-surface electric field is closely related to the physical structure of the AuNBr, it can be understood that with the properly added AA volumes in the reaction mixture, the prepared AuNBr with optimized core size (30 nm) and branches (a = b = 7 nm, c = 10 nm) can produce the strongest near-surface electric field intensity. In view of the approximated fourth power relation (|*E*|^4^) between the Raman intensity and the electromagnetic field, the AuNBr synthesized with 40 μL AA ultimately gives the best ***EF*** as shown in Table 2.

## 5. Conclusions

In all, we have prepared AuNBr with an efficient one-pot synthesizing method, while using no further chemicals except HAuCl_4_, AA and HEPES. In this facile synthetic procedure, the dependence of the core size and branches on the AA volumes is comprehensively investigated, and a possible growing mechanism of the AuNBr is proposed. Moreover, by using the 4-MBA as the reporter, the SERS performance is evaluated; results show that the optimized AuNBr shows superior SERS activity and can be used for future bio-sensing applications.

## Figures and Tables

**Table 1:** The detailed structural parameters of the synthesized AuNBr measured from TEM images (see Figure S1 in supplementary).

AA/μL	Mean Radius of Core/nm	Mean Branches/nm ^1^	
		a = b	c
10	50 ± 4.1	5 ± 1.3	10 ± 2.2
20	40 ± 4.0	5.5 ± 1.5	10 ± 2.3
30	35 ± 3.1	6 ± 1.8	10 ± 2.2
40	30 ± 3.5	7 ± 1.8	10 ± 2.3
50	30 ± 3.2	7 ± 2.2	7.5 ± 1.7
60	26 ± 3.0	7 ± 2.1	7 ± 1.8
70	25 ± 2.9	7 ± 1.8	6 ± 1.5
80	25 ± 2.8	7 ± 2.3	4 ± 1.6

^1^ The equation of the ellipsoid is $\frac{x^{2}}{a}+\frac{y^{2}}{b}+\frac{z^{2}}{c}=1$.

**Table 2:** The calculated enhancement factors of the synthesized AuNBr with different amounts of AA volumes.

	AA/μL	*EF*
Sample 1	10	6.6 × 10^7^
Sample 2	20	9.3 × 10^7^
Sample 3	30	9.6 × 10^7^
Sample 4	40	1.5 × 10^8^
Sample 5	50	8.5 × 10^7^
Sample 6	60	5.2 × 10^6^
Sample 7	70	4.8 × 10^6^
Sample 8	80	2.7 × 10^6^
Au nanostar (ref. [19])	-	~10^7^
Au nanopolyhedral (ref. [27])	-	~10^5^ to 10^6^
Au nanorod (ref. [35])	-	~10^8^
Au nanoflower (ref. [36])	-	~10^8^
Au nanourchins(ref. [37])	-	~10^9^

## Supplementary Materials

The following are available online at http://www.mdpi.com/2079-6374/8/4/113/s1. Figure S1: During the measurement, the uniformly dark solid area is considered as core, the grey and translucent part is considered as the branch, and those nanoparticles with extremely abnormal size are ignored. The structural parameters of the AuNBr are shown (a) 10 μL; (b) 20 μL; (c) 30 μL; (d) 40 μL; (e) 50 μL; (f) 60 μL; (g) 70 μL; (h) 80 μL. The red crosses indicate the branches that are measured, Figure S2: The FEM simulated absorption spectra and near-surface electric field intensity for the nanoparticles synthesized with the AA of (a) 20 μL; (b) 70 μL. The electric field is extract under the irradiation wavelength of 785 nm.
