# Supplementary material for: One-Pot Synthesis of Multi-Branch Gold Nanoparticles and Investigation of Their SERS Performance
Source: Biosensors (Basel). 2018 Nov 20;8(4):113. doi: 10.3390/bios8040113 (PMC6315941; doi:10.3390/bios8040113)
Supplement: Supplementary file 1 [file biosensors-08-00113-s001.pdf]

Supplementary Material: One-pot Synthesis of Multi-branch Gold Nanoparticles and Investigation on Their SERS Performance

Weifeng Lv [1], Chenjie Gu [1], Shuwen Zeng[2,*], Jiaguang Han[3], Tao Jiang[1] and Jun Zhou [1,*]

[1] Institute of Photonics, Faculty of Science, Ningbo University, Ningbo 315211, Zhejiang, China
[2] XLIM Research Institute, UMR 7252 CNRS/University of Limoges, 87060 Limoges CEDEX, France
[3] Center for Terahertz Waves and College of Precision Instrument and Optoelectronics Engineering, Tianjin University and the Key Laboratory of Optoelectronics Information and Technology (Ministry of Education), Tianjin 300072, China
* Correspondence: zhoujun@nbu.edu.cn (J.Z.); shuwen.zeng@unilim.fr (S.Z.); Tel.: +86-574-8760-0794 (J.Z.); +33-555-457-532 (S.Z.)

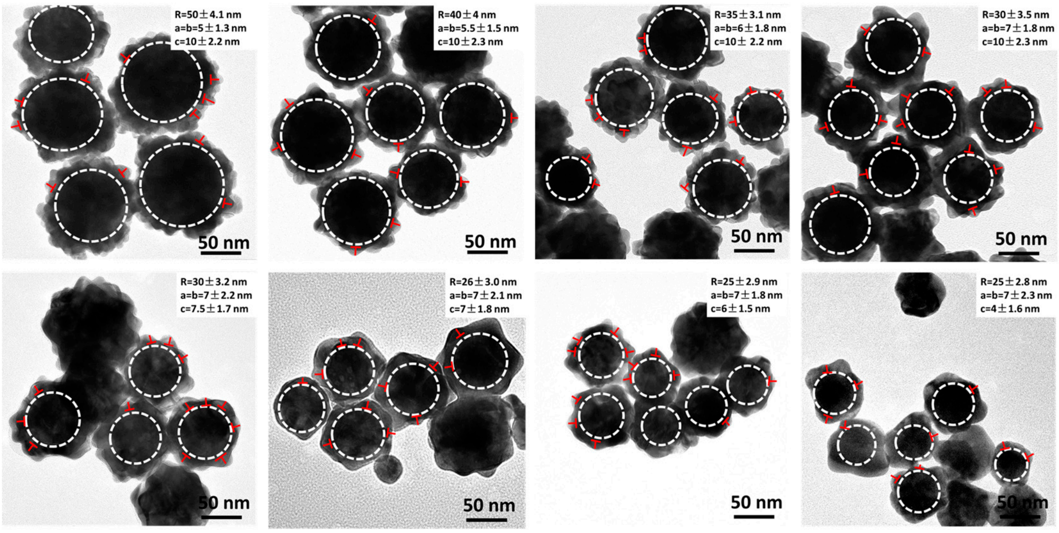

Figure S1. During the measurement, the uniformly dark solid area is considered as core, the grey and translucent part is considered as the branch, and those nanoparticles with extremely abnormal size are ignored. The structural parameters of the AuNBr are shown (a) 10 μL; (b) 20 μL; (c) 30 μL; (d) 40 μL; (e) 50 μL; (f) 60 μL; (g) 70 μL; (h) 80μL. The red crosses indicate the branches that are measured.

Calculating the SERS enhancement factors (*EFs*)

The SERS enhancement factors are calculated by the following equation:

$$\boldsymbol{EF} = (\boldsymbol{I_{SERS}} \times \boldsymbol{N_{NR}})/(\boldsymbol{I_{NR}} \times \boldsymbol{N_{SERS}})$$

where I_{SERS} and I_{NR} are the integrated peak intensities at 1078 cm^{-1} from SERS and from bulk Raman, respectively. N_{SERS} is the number of molecules probed in the bulk sample, and N_{NR} is the number of molecules adsorbed on the nanoparticles. Here we will use the Raman spectrum of 4-MBA solution (10 mM) to evaluate the N_{NR} in the bulk. Based on our measurement setting, the diameter of illumination focus is first calculated by using the following equation [1]:

$$D_{illumination} = \frac{1.22\lambda}{N.A.} \ldots\ldots Eq. (1)$$

in which the ***N.A.***= 0.65 the Raman spectrometer at the 785-nm wavelength. In addition, the penetration depth (h) of laser beam in the bulk 4-MBA is about 5.12 mm, thus it gives the

$$N_{bulk} = \frac{1}{3}\pi\left(\frac{D_{illumination}}{2}\right)^2 hcN_A \ldots\ldots Eq. (2)$$

Where c is the concentration of 4-MBA solution (10 mM), N_A is the Avogadro constant. The above equation ultimately gives the $N_{bulk} = 1.75 \times 10^{10}$

To calculate the N_{SERS}, 20μL of 10 mM 4-MBA solution is added into 3 mL ($V_{solution}$) of Au nanoparticle colloidal solution in the quartz cuvette, forming the 4-MBA labeled Au nanoparticle, then the excitation light of 785-nm wavelength is directly irradiated on the colloidal solution to collect the SERS spectra. The effective excitation depth is assumed as a monolayer of nanoparticle in the colloidal solution. The effective excitation volume is

$$V_{SERS} = \pi\left(\frac{D_{illumination}}{2}\right)^2 R_{particle} \ldots\ldots Eq. (3)$$

Where $R_{particle}$ is averaged radius of the nanoparticle. The number of the molecules adsorbed on the nanoparticles can be approximated by the volume ratio as follows:

$$N_{SERS} = V_{SERS} \times \frac{N_{solution}}{V_{solution}} \ldots\ldots Eq. (4)$$

Based on *Eq. (1)* to *Eq. (4)*, we have the N_{SERS} for the cases in the experiment as follows:

	N_{SERS}	I_{SERS}	*EF*
Sample 1	4.088×10^3	1.66281×10^5	6.6×10^7
Sample 2	3.404×10^3	1.94842×10^5	9.3×10^7
Sample 3	3.064×10^3	2.01603×10^5	9.6×10^7
Sample 4	2.724×10^3	2.54540×10^5	1.5×10^8
Sample 5	2.552×10^3	1.35489×10^5	8.5×10^7
Sample 6	2.248×10^3	7.2762×10^4	5.2×10^6
Sample 7	2.112×10^3	6.2672×10^4	4.8×10^6
Sample 8	1.960×10^3	3.3040×10^4	2.7×10^6

$* I_{NR} = 1.0813 \times 10^4$

[1] R.A. Álvarez-Puebla, Effects of the excitation wavelength on the SERS spectrum, J. Phys. Chem. Lett. 3 (2012) 857–866.

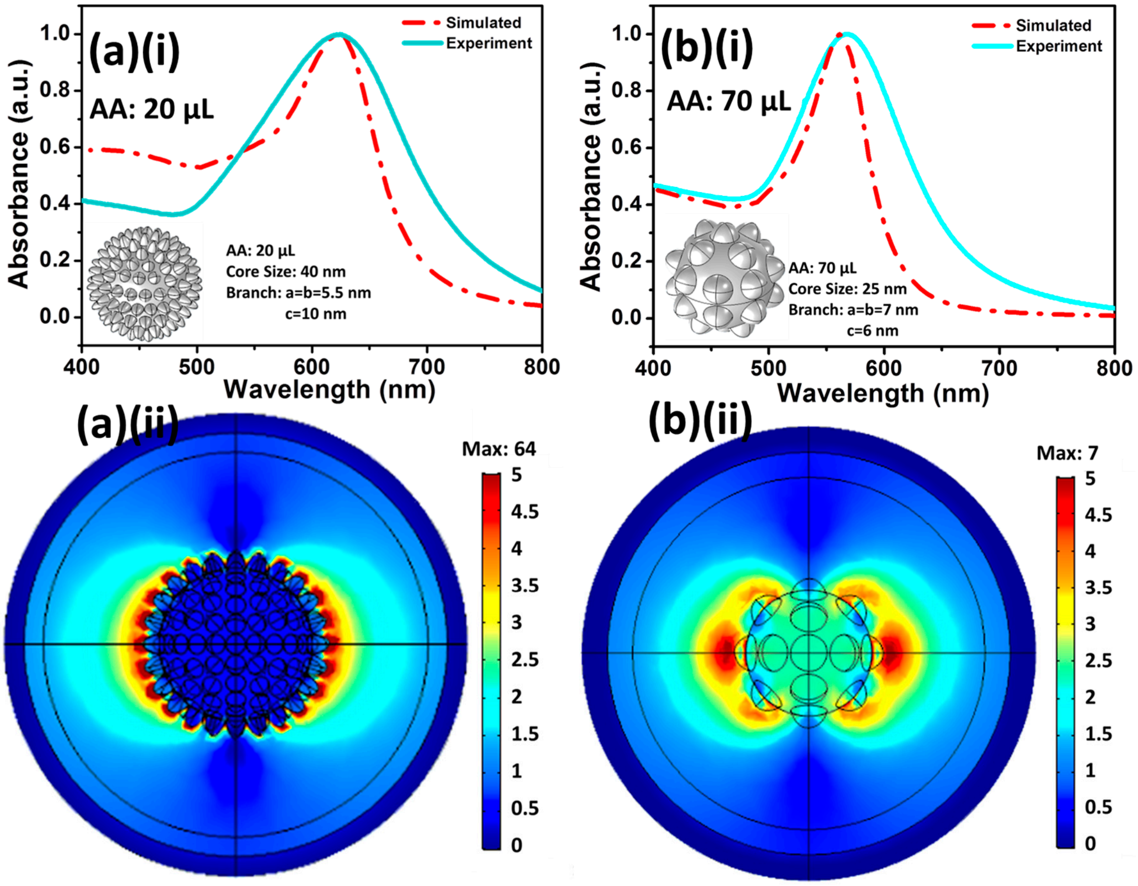

Figure S2. The FEM simulated absorption spectra and near-surface electric field intensity for the nanoparticles synthesized with the AA of (a) 20 μL; (b) 70 μL. The electric field is extract under the irradiation wavelength of 785 nm
